# Experiences of children with trisomy 18 referred to pediatric palliative care services on two continents

**DOI:** 10.1002/ajmg.a.61149

**Published:** 2019-04-01

**Authors:** Jonathan Mullin, Joanne Wolfe, Myra Bluebond‐Langner, Finella Craig

**Affiliations:** ^1^ Department of Pediatrics Washington University in St Louis St. Louis Missouri; ^2^ Department of Psychosocial Oncology and Palliative Care Dana‐Farber Cancer Institute Boston Massachusetts; ^3^ Department of Medicine Boston Children's Hospital Boston Massachusetts; ^4^ Faculty of Population Health Sciences, Louis Dundas Centre for Children's Palliative Care UCL Great Ormond Street Institute of Child Health London United Kingdom; ^5^ Louis Dundas Centre for Children's Palliative Care Great Ormond Street Hospital for Children NHS Foundation Trust London United Kingdom

**Keywords:** palliative care, pediatrics, trisomy 18

## Abstract

Children with trisomy 18 that survive beyond the neonatal period have multiple congenital anomalies, neurodevelopmental disability, and high mortality rates. The experience of children with trisomy 18 who receive pediatric palliative care services is largely unknown. We conducted a retrospective review of children with trisomy 18 receiving pediatric palliative care services at both Boston Children's Hospital, USA and Great Ormond Street Hospital, UK from January 1, 2004 to January 1, 2015. Fifty‐eight children with trisomy 18 were referred to pediatric palliative care, 38 in the United Kingdom, 20 in the United States. Median age at referral was 19 days (2–89) in the United Kingdom, and 25 days (1–463) in the United States. Median length of time being followed by pediatric palliative care was 32 days (1–1,637) in the United Kingdom and 67 days (3–2,442) in the United States. The only significant difference in the two cohorts (*p* = .001) was in likelihood of receiving cardiac surgical intervention—37% in the United States, 0% the United Kingdom. Children with trisomy 18 receive pediatric palliative care services, with variable age at referral and for a variable length of time. Further research is needed to understand the experience of children with trisomy 18 and their families receiving pediatric palliative care services.

## 1 INTRODUCTION

Trisomy 18 is the second most common autosomal numerical chromosomal disorder, occurring in 15 of every 100,000 live births (Parker et al., 2010) and leads to multi‐organ system anomalies, including cardiac malformations and neurological impairment (Merritt et al., 2012). Population studies have shown that over 50% of children born with trisomy 18 die within weeks of being born, and less than 10% survive beyond 1 year (Irving, Rochmond, Wren, Longster, & Embleton, 2011; Meyer et al., 2015; Rasmussen, Wong, Yang, May, & Friedman, 2003; Vendola et al., 2009). Children that live into early childhood have neurological impairment, with significant cognitive, language, and motor delays (Lorenz & Hardart, 2014; Meyer et al., 2015). However, recent longitudinal studies have shown 5‐ and 10‐year survival rates of 10–13% (Meyer et al., 2015; Nelson, Rosella, Mahant, & Guttmann, 2016) and these children have increasing hospitalizations and procedural interventions (Nelson, Hexem, & Feudtner, 2012). Increasing data about survival, interventions, and health care utilization have challenged previous convention that trisomy 18 is uniformly lethal, not “compatible with life,” and that interventions are futile, and should not be offered (Janvier & Watkins, 2013; McCaffrey, 2016).

Pediatric palliative care (PPC) is child‐ and family‐centered and focuses on relief of suffering and enhancing quality of life for children with chronic, serious, or life‐threatening conditions (Himelstein, Hilden, Boldt, & Weissman, 2004). A majority of children receiving PPC have a genetic diagnosis and/or congenital malformation, most have some degree of cognitive impairment, and are followed typically by PPC teams for at least 1 year (Feudtner et al., 2011). Despite significant overlap in the characteristics of children with trisomy 18 and children who receive PPC services, the experience of children with trisomy 18 receiving PPC services is largely unknown. Studies have focused primarily on perinatal palliative care referrals with short‐term survival data and follow up (Marc‐Aurele, Hull, Jones, & Pretorius, 2018). The primary aim of this study was to describe the experience of children with trisomy 18 receiving PPC services at two large tertiary care pediatric centers on different continents.

## 2 METHODS

This study was approved by the Institutional Review Board at Boston Children's Hospital (BCH), and registered with the Clinical Audit Department at Great Ormond Street Hospital (GOSH). We conducted a retrospective chart review of children with the diagnosis of trisomy 18 referred to PPC Consultative Services from January 1, 2004 to January 1, 2015 at Boston Children's Hospital, Boston, MA and Great Ormond Street Hospital, London, UK. PPC at both institutions includes inpatient, outpatient, and perinatal consultation. All children with the diagnosis of trisomy 18 referred to PPC, and for whom a PPC consultation was performed were included. Children with partial or mosaic trisomy 18 were also included. Children lost to follow up were counted as alive with length of life based on date of last follow up at each hospital. All data were obtained from medical records, information regarding surgical intervention was based on procedure notes. Surgical anomalies, both cardiac and non‐cardiac, were defined as any anomaly that would typically require surgical intervention, not whether surgery was indicated or performed. Cardiac surgical intervention was defined as any procedure performed by a cardiac surgeon or interventional cardiologist, including open procedures, those performed via catheterization, and other less invasive techniques.

Descriptive statistics were used to describe the study cohort by location. Comparisons between groups were analyzed with Fisher's exact test for categorical variables and Mann–Whitney *U* test for continuous variables. Kaplan–Meier survival curves were created for cohorts at both locations and were compared with a log‐rank test. *p* Values less than .05 were considered significant.

## 3 RESULTS

Fifty‐eight children with trisomy 18 were referred to PPC services, 38 at GOSH, 20 at BCH. Eleven of the referrals at GOSH were antenatal, five died prior to being born, and there were no antenatal referrals at BCH. The majority of children referred were female, 79% percent at GOSH, and 75% at BCH (Table 1).

**Table 1:** Patient characteristics

	Total *N* = 58	GOSH *N* = 38	BCH *N* = 20	*p*‐Value
Antenatal referrals, no (%)	11 (19)	11 (29)	0 (0)	
Live births, no (%)	53 (91)	33 (87)	20 (100)	
Female (of live births), no (%)	41 (77)	26 (78)	15 (75)	.748
Median age (days) at postnatal consult, no (range)	19 (1–463)	18 (2–89)	25 (1–463)	.267
Alive at time of study, no (%)	13 (23)	7 (21)	6 (30)	.533
Median age (days) of surviving patients, days (range)	599 (104–2,505)	599 (151–1,637)	801 (104–2,505)	.834
Median age at death (days), no (range)	48 (1–830)	459 (1–142)	55 (37–830)	.930
Median time (days) followed by palliative care (range)	56 (1–2,442)	32 (1–1,637)	67 (3–2,442)	.107
Cardiac anomaly, no (%)	48 (91)	29 (88)	19 (95)	.639
Underwent cardiac surgical intervention, no (%)	7 (13)	0 (0)	7 (37)	.001
Non‐cardiac surgical anomaly, no (%)	14 (26)	8 (24)	6 (30)	.751
Underwent non‐cardiac surgical intervention, no (%)	8 (57)	3 (38)	5 (83)	.138

Abbreviations: BCH, Boston Children's Hospital; GOSH, Great Ormond Street Hospital.

Median age at time of PPC consult was 19 days (range: 2–89) at GOSH, and 25 days (1–463) at BCH. Median length of time being followed by PPC was 32 days (1–1,637) at GOSH and 67 days (3–2,442) at BCH. Of the children who died, median age of death was 49 days (1–142) at GOSH and 55 days (37–830) at BCH. Median age of surviving patients was 599 days (151–1,637) at GOSH and 801 days (104–2,505) at BCH.

**Figure 1:**
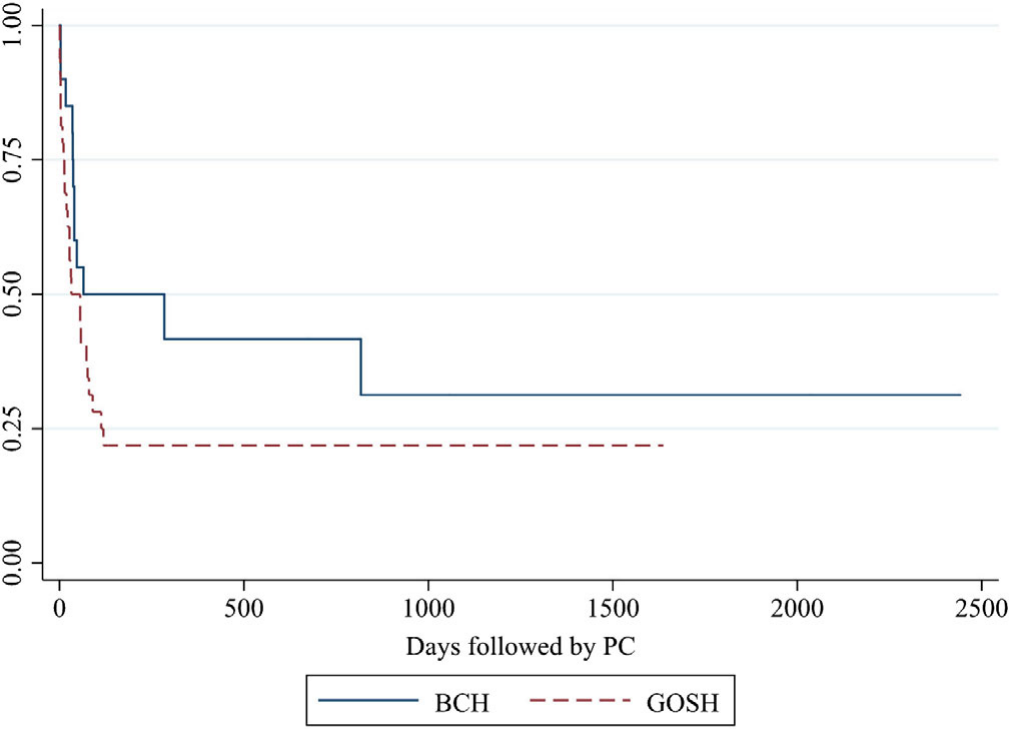
Survival of children with trisomy 18 followed by pediatric palliative care Abbreviations: BCH, Boston Children's Hospital; GOSH, Great Ormond Street Hospital [Color figure can be viewed at wileyonlinelibrary.com]

Children with trisomy 18 lived from 0 to 2,505 days. At the time of study, seven were alive at GOSH, five were alive at BCH. No children were lost to follow‐up at GOSH, two were lost to follow up at BCH. Survival curves of each cohort are presented in Figure 1. There was no significant difference in survival (*p* = .095).

A majority of children at both institutions had a congenital heart anomaly, 88% at GOSH, 95% at BCH. Of the children with congenital heart disease, none underwent cardiac surgery at GOSH, while seven (37%) underwent cardiac surgery at BCH (*p* = .001). A minority of children had a non‐cardiac surgical anomaly, 21% at GOSH and 30% at BCH, and of those children, three (38%) at GOSH and five (83%) at BCH underwent a surgical procedure.

## 4 DISCUSSION

We report data from children with trisomy 18 who received PPC services in large tertiary care centers, on two different continents. These children were referred to PPC predominantly in the first few weeks of life, or antenatally. Variation in prevalence of antenatal consults likely reflects differences in institutional referral patterns. Several children underwent surgical interventions, for both cardiac and non‐cardiac anomalies, predominantly at the U.S. institution. This difference may reflect differing parental preference around intervention, institutional precedents for or against cardiac intervention for children with trisomy 18, and differences in patient characteristics that led to PPC referral.

Survival data for children with trisomy 18 who received PPC services mirrors that of larger population studies (Nelson et al., 2016) where the majority die within weeks, but a smaller percentage live for years. The trend toward longer survival at BCH, may be indicative of differing rates of cardiac surgical interventions, which has been shown to be associated with increased survival in cohorts of children with trisomy 18 in Japan (Kaneko et al., 2009; Maeda et al., 2011).

Although not directly assessed during this study, our experiences suggest the reason for referral of a child with trisomy 18 to PPC, as well as the role of PPC in the care of children with trisomy 18 is variable. Referrals may be made based on diagnosis alone, for decisional support when interventions are being considered, for assistance with transition of care to home or hospice, or as children live “longer than expected” they may be referred to PPC explicitly because they have survived. The amount of time children with trisomy 18 were followed by PPC services was also variable, likely determined by survival, and reflective of the varied role that consults fulfilled, ranging from support and symptom management at end‐of‐life, to providing palliative care (revisiting goals of care, psychosocial support, medical decision‐making support, ongoing symptom management, transitioning to end‐of‐life care, etc.) longitudinally throughout these children's lives.

Predictors of survival for children with trisomy 18 are largely unknown. Multiple studies on intensive medical management in the neonatal period, surgical interventions, and cardiac medical and surgical interventions have been mixed, but seem to show improvements in survival for children with trisomy 18 (Bruns & Martinez, 2015; Costello et al., 2015; Donovan, Krigbaum, & Bruns, 2016; Janvier, Farlow, & Barrington, 2016; Kaneko et al., 2008; Kosho et al., 2006; Kosiv, Gossett, Bai, & Collins, 2017; Nakai, Asano, Nomura, Matsumae, & Mishima, 2016; Nelson et al., 2016; Nishi et al., 2013; Peterson, Calamur, Fiore, Huddleston, & Spence, 2018; Peterson, Kochilas, Catton, Moller, & Setty, 2017; Subramanian et al., 2015). However, it is unclear if children with trisomy 18 are living longer because of surgical interventions offered, or if children who are likely to live longer (because they are older or healthier) are offered the interventions. Review of the literature suggests that consideration of intensive medical or surgical intervention should be conducted on a case‐by‐case basis, with attention to family preferences and assessment of quality of life (Costello et al., 2015; Donovan et al., 2016; Janvier et al., 2016; Kosho & Carey, 2016), appreciating that clinicians and parents do not necessarily conceptualize quality of life in the same way (Beecham, Langner, Hargrave, & Bluebond‐Langner, 2019).

Trisomy 18 is increasingly recognized as a heterogeneous condition (Janvier et al., 2016) and the same applies to those children with trisomy 18 receiving PPC services. Attitudes of providers toward the care of children with Trisomy 18 vary, likely related to the characteristics of the subpopulation of children for which they care (Fruhman et al., 2018; Hurley, Krishnan, Parton, & Dozor, 2014; Yates, Hoffman, Shepherd, Boettner, & McBride, 2011; Young, Simpson, & Warren, 2017). Although there have been no formal studies of attitudes of PPC providers, some have endorsed a patient‐centered and shared decision‐making model of care (Boss et al., 2013; Brosco & Feudtner, 2017). This approach may be influenced by the variability in course and survival of the subpopulation of children with trisomy 18 cared for by PPC providers.

This study has several limitations. Patient sample sizes were small and limited to two institutions. PPC services are dependent on referrals from other providers, and thus the study population represents only those patients deemed eligible for PPC involvement, for reasons that may extend beyond patient characteristics (e.g., challenging psychosocial situations, difficulty establishing goals of care, challenging symptoms). We did not assess reasons for PPC referral, nor was this study able to evaluate the impact of PPC on survival or surgical interventions. We did not compare specific practices of PPC at each institution, which could have contributed to differences in surgical interventions pursued. However, both PPC teams support shared medical decision making between medical and surgical teams and the family and thus we believe the difference in surgical interventions is most likely due to differences in institutional standards of care and family preference. In addition, this study included children with partial or mosaic trisomy 18. Although these children may have a less severe phenotype, and could therefore impact survival data, this subgroup of children is small in number, and has minimal effect on the data.

To our knowledge, this is the first published data about the longitudinal experience of children with trisomy 18 followed by PPC services. This study calls for further investigation into the longitudinal experience and care provided to children with trisomy 18, and how their care may differ from the care of children with similar genetic conditions, based on experiences of having a diagnosis previously thought to be “incompatible with life” (Janvier, Farlow, & Wilfond, 2012). Important questions include optimal timing of PPC referral, and the role of antenatal PPC consults for children with trisomy 18. As care for children with trisomy 18 expands beyond the delivery room and the neonatal intensive care unit, the role of early PPC involvement in these children's care, as well as family values, preferences, and approaches to decision making also merit exploration.

Given the shifting approach to the care of children with trisomy 18, and the role of PPC in the care of children with chronic and life‐limiting diagnoses, PPC providers will likely be caring for an increasing number of children with trisomy 18. This study provides a glimpse into this patient population, which is anticipated to continue growing, and warrants closer investigation in the years to come.

### DATA ACCESSABLITY

Data available on request due to privacy/ethical restrictions.
